# Comparative Performance of Kevlar, Glass and Basalt Epoxy- and Elium-Based Composites under Static-, Low- and High-Velocity Loading Scenarios—Introduction to an Effective Recyclable and Eco-Friendly Composite

**DOI:** 10.3390/polym16111494

**Published:** 2024-05-24

**Authors:** Farid Taheri, Jesse R. J. G. Llanos

**Affiliations:** Advanced Composites and Mechanics Laboratory, Department of Mechanical Engineering, Dalhousie University, Halifax, NS B3H 4R2, Canada; js205258@dal.ca

**Keywords:** Elium, basal composite, mechanical properties, high-velocity impact, low-velocity impact, impact response, eco-friendly composites

## Abstract

In general, the majority of fiber-reinforced polymer composites (FRPs) used in structural applications comprise carbon, glass, and aramid fibers reinforced with epoxy resin, with the occasional utilization of polyester and vinyl ester resins. This study aims to assess the feasibility of utilizing recyclable and sustainable materials to create a resilient composite suitable for structural applications, particularly in scenarios involving low-velocity and high-velocity impact (LVI, HVI) loading. The paper presents a comparative analysis of the performance of E-glass, aramid, and eco-friendly basalt-reinforcing fabrics as reinforcement fibers in both thermosetting (epoxy) and recyclable thermoplastic (Elium^©^) resins. Given the limited research on Elium composites, especially those incorporating basalt-reinforcing fiber, there is an urgent need to expand the databases of fundamental mechanical properties for these diverse composites. This necessity is exacerbated by the scarcity of the literature regarding their performance under low- and high-velocity impact loadings. The results of this study will demonstrate the potential of basalt-reinforced Elium composite as an effective recyclable and environmentally friendly structural material system for both static and dynamic loading conditions.

## 1. Introduction

Currently, most fiber-reinforced polymer composites (FRPs) used in demanding structural applications predominantly utilize carbon and glass as well as aramid fibers reinforced with epoxy resin, though, occasionally, polyester and vinyl ester resins are also used. However, one of the significant drawbacks of the use of thermoset resins is their brittle and unrecyclable nature, which generates plastic waste during their fabrication and at the end of their lifecycle, in turn causing significant environmental impediments. Their brittle nature makes them susceptible to damage, especially when subjected to an impact, which in most cases could be invisible to the naked eye, thus compromising their structural integrity.

In general, fiber-reinforced thermoplastics have been widely used in several engineering applications such as in turbine blades and the automotive and aerospace industries [1]. Elium is the world’s first low-viscosity liquid thermoplastic resin and is fully recyclable. With its relatively higher ductility and toughness, Elium has created an opportunity to develop enhanced FRPs and address the aforementioned issues. The full recyclability of Elium was demonstrated by the ZEBRA (Zero wastE Blade ReseArch) consortium in France [2] when the first sample of its 100% recyclable 77 m long E-glass–Elium wind turbine blades was completed. The blade was fabricated in Spain and produced a 15% increase in energy efficiency with no modification to the base structure. However, due to its chemical nature, Elium is limited to manufacturing techniques that use low-pressure processes like vacuum-assisted resin infusion molding (VARTM) or resin transfer molding (RTM). Its recyclable nature could also potentially reduce the waste associated with thermosetting plastics. Elium’s ductility also has the potential to enhance the impact resistance of its composites, leading to longer service life and increased part durability.

Compared to the available research on various thermoset and thermoplastic composites, however, research specifically addressing composites made with Elium resin is relatively limited. This gap becomes even more apparent when considering studies that have explored Elium composites using fibers other than the conventionally used glass and carbon fibers (e.g., fibers such as Kevlar and particularly strong and stiff eco-friendly fibers like basalt fibers). This fact motivated the experimental investigation presented in this study.

As for reinforcing fibers, basalt has garnered considerable attention lately. It is a naturally occurring substance made of a type of igneous rock, with a composition that is approximately 50% silica, 18% aluminum oxide, and 10% iron oxide, along with other trace minerals [3]. Since basalt is naturally occurring, the processing required to produce basalt fibers is significantly smaller when compared to other available fibers, making it a more economical alternative. Moreover, its waste would have a significantly smaller impact on the environment, especially if the fibers were paired with a recyclable matrix. Its smaller environmental footprint gives basalt the potential to be implemented as an eco-friendly replacement for existing fiber reinforcement materials. Basalt is manufactured by first raising the substance to its melting point and then extruding the melt products to produce the fiber. Basalt fibers have also been used as an insulating material, because of their thermal properties, and as a structural material, since their mechanical properties are comparable to other available reinforcing fibers.

Even the current studies that have investigated Elium composites have mostly considered glass and carbon composites under low-velocity impact as the critical loading state. Therefore, there is a clear need for a systematic investigation to establish the basic mechanical properties of Elium-based FRPs made with different reinforcing fibers, especially with mineral-based fibers such as basalt. Moreover, the extension of the database regarding their performance under static and critical loading states such as low- and high-velocity impact scenarios would be a useful task.

In summary, the work presented here entails the experimental characterization of the basic mechanical properties, as well as both the low-velocity impact (LVI) and high-velocity impact (HVI) responses, of composites fabricated using Elium 150 thermoplastic resin and three different fibers (i.e., E-glass, aramid, and basalt). The results from these experiments will be compared to their epoxy-based counterparts. Moreover, the viability of Elium-basalt composite as an eco-friendly and recyclable composite for structural applications will also be discussed. The results produced in this study are believed to be unique in the available literature and should be useful for designers of lightweight structural systems.

## 2. Literature Review

Elium resin, a type of thermoplastic liquid acrylic resin, was developed relatively recently (2009) by Arkema of France. This resin is the world’s first thermoplastic liquid resin that cures at room temperature. Similar to epoxy, Elium has low viscosity, which is chemically crosslinked by radical polymerization where its monomer, methyl methacrylate (MMA), transitions to its polymer form PMMA through the use of a peroxide catalyst [4,5].

It should be noted that the volume of FRPs made of thermoset resin significantly surpasses those made of thermoplastics, mainly due to the unavailability of room-cured thermoplastic. Moreover, processing techniques such as resin transfer molding, infusion, pultrusion, and filament finding would be impossible with traditional thermoplastics, which are usually available in the form of pellets or films. Therefore, the emergence of Elium has attracted considerable attention. Since its introduction, Elium has been successfully used in fabricating large and small structural components and systems such as wind turbine blades and small and large boats [6,7,8].

While the performance of various Elium composites has been investigated by several research groups, the volume of research and the size of the database representing the performance of such composites are still dwarfed compared to thermoset-based FRPs. Some of the notable relevant research on Elium composites is presented below.

The performance of Elium flax fabric-reinforced Elium and epoxy composites under the influence of water was characterized by Chilali et al. [9], incorporating monotonic and load–unload cyclic tests. The investigation concluded that the two composites responded similarly to their water-aging environment and the subsequent cyclic mechanical testing. The results of their study showed that the two composites had approximately similar variations in their elastic and failure properties after aging in a wet environment (i.e., approximately 10% degradation in the tensile stiffness when subjected to cyclic testing). Bhudolia and Joshi [10] investigated the low-velocity impact performance of Elium and epoxy matrices reinforced with carbon fiber at three energy levels (25 J, 42 J, and 52 J). The results indicated that the Elium composite underwent higher elastic deflection up to its ultimate capacity. The lower residual deflection (−53%) showed an improvement in the structural integrity compared to its epoxy composite counterpart. Also, it was noticed that a significant amount of energy (58%) was absorbed by the Elium composite before the onset of failure, mostly through elastic–plastic deformations.

Barbosa et al. [11] investigated the performance of basalt–Elium against basalt–epoxy laminates and revealed that the Elium-based composite exhibited a 5% lower stiffness but 23.5% higher tensile strength compared to the equivalent epoxy-based composite. However, when these values were normalized, the 5% difference vanished, and the tensile strength of the Elium-based composite was shown to be 28.4% superior to the epoxy-based composite. Barbosa et al. [11] focused on evaluating the out-of-plane properties of the (0/90) plain-weave carbon fabric-reinforced Elium composite compared to its epoxy counterpart. The results showed that the carbon–Elium laminate composite produced a higher resistance to crack propagation. Additionally, although its failure occurred at a lower energy level (125.25 J/m^2^) in the non-pre-cracked state compared to its thermoset composite counterpart (195.10 J/m^2^), it could resist up to 40% (214.22 J/m^2^) more energy than the epoxy composite (201.22 J/m^2^) in the pre-cracked state. Nash et al. [6] investigated the effect of environmental conditions on the performance of glass fiber-reinforced polymer laminates made of a range of thermosetting resins, including vinyl ester, polyester, epoxy, and Elium for marine applications using the resin infusion technique. The results showed that their epoxy composite absorbed 24% more water than the Elium composite. In all, the vinyl ester and polyester composites exhibited the lowest water absorption among the composites, and their values were similar and almost half of that of the epoxy composite. Moreover, vinyl ester and Elium composites had the highest and lowest absorption of diesel fuel, respectively.

It is well known that the through-thickness performance is governed by the fracture toughness of resin and fibers. Bhudolia et al. [12] demonstrated that the incorporation of a thermoplastic while fabricating FRPs resulted in significant improvement. They investigated the fracture toughness of composites fabricated using three different fibers and Elium resin. They observed the highest mode II fracture toughness for the composites constructed with ultra-high-molecular-weight polyethylene fibers reinforced with Elium. In another study, Barbosa et al. [11] conducted similar research and demonstrated the significantly higher fracture toughness of carbon–Elium (+40%) in comparison to its carbon–epoxy equivalent. Another study conducted by Bhudolia et al. [13] revealed a similar flexural response between Elium– and epoxy–carbon FRPs. Additionally, they showed the annealing of Elium, which increased the polymerized resin sites, resulting in a 21% and 11% higher flexural strength and modulus compared to the laminate with no annealing.

In general, while several studies have shown that the mechanical properties of Elium-based composites are quite similar to those of common thermoset resin composites [4,14], Elium composites’ resin–fiber interface strength would be somewhat affected by the relatively poor resin–fiber interface strength of Elium compared to its thermoset counterparts.

Another positive attribute of Elium composite is its superior adhesion to other materials, such as cores in sandwich composites. An example of this attribute has been demonstrated by Alshahrani et al. [15]. They investigated the flatwise tensile, flexural, and climbing drum peel strengths of sandwich composites made of glass–epoxy and glass–Elium with PVC and PET foams. They observed the Elium-based PVC sandwich core to be significantly better than the epoxy-based sandwich under flexural and tensile loadings. Moreover, the Elium-based sandwich offered the highest peel strength, which was 53% greater than its epoxy-based counterpart. The results demonstrated the superior interface bonding characteristics of Elium compared to epoxy.

As stated earlier, thermoplastics have an advantageous attribute of being recyclable, though recycling comes with a few potential drawbacks like void formation, impurity inclusion, and weakening properties in the crystal structure. However, one can reduce such drawbacks significantly by incorporating effective quality control. The recyclability of flax–Elium composites was investigated by Allagui et al. [16], who observed a decrease in the tensile properties of the composite as a result of recycling. Their further analysis revealed that the change in the elastic properties was mainly due to the reduction in the fiber size, which resulted from the incorporated recycling approach. Allagui et al.’s investigation was further expanded by Sahki [17], who investigated the influence of recycling methods on E-glass–Elium and basalt–Elium composites. Her test results showed minimal differences in the properties between glass and basalt composites. She also achieved the complete recycling of the resin with minimal loss in the properties of the recycled composite by using solvolysis/dissolution [17].

It should be noted that the recycling of the resin, which is facilitated by the weakened van der Waals interactions as a result of heating/melting thermoplastic resins, also offers the ability of the cured resin to be welded to itself. Bhudolia et al. [18] investigated the fatigue response of the ultrasonically welded carbon/Elium composites by comparing them to adhesively bonded joints. They observed an increase in fatigue strength in the range of 7–12%.

Elium composites also offer improved vibration-damping characteristics compared to epoxy-based composites. This was verified by another study conducted by Bhudolia et al. [19], who observed a 27% increase in the structural damping of Elium-based composites compared to epoxy-based composites.

The impact characteristics of Elium composites have also been investigated by researchers; however, the volume of research concerning the low-velocity impact (LVI) of Elium composites is relatively quite limited and even more limited when considering HVI-related studies. It should be noted that when laminated composites are subjected to an impact loading, the energy is absorbed through several mechanics, including deformation delamination, fiber breakage, and matrix failure [20,21,22]. Bhudolia and Joshi [10] investigated the low-velocity impact response of a carbon–Elium composite against a carbon–epoxy composite. The Elium-based composite was observed to undergo 53% higher elastic deformation than its epoxy counterpart. Moreover, the authors observed comparatively 58% more absorbed energy by the Elium-based composite before the onset of a major failure, which was facilitated by a significant elastic–plastic deformation response. The performances of helmets made of carbon–epoxy and carbon–Elium composites by Gohel et al. [22] revealed the superior energy absorption capacity and impact energy dissipation capability of the carbon–Elium composite, which was attributed to the improved toughness of the Elium resin.

Kazemi and his co-workers have also conducted several studies investigating the LVI response of Elium-based composites. In one of their studies [23], they observed a remarkable increase of 240% in the structural integrity of their post-impact specimens of Elium–carbon composite compared to carbon–epoxy composite. Several other similar investigations have also been reported by other researchers [10,23,24,25,26,27]. However, as mentioned previously, there is a clear lack of HVI-related investigation into Elium-based composites compared to the commonly used epoxy-based composites. One such study is that by Libura et al. [28], who investigated the effect of fatigue and aging on the ballistic limit of woven glass-reinforced Elium laminates. They found that fatigue and aging deteriorated the fiber/matrix interface, thus leading to a reduction in the stiffness and ballistic performance of the composite.

Since some of the most prominent and positive attributes of Elium compared to its brittle thermoset counterpart resins are its ductility and toughness, this resin is expected to perform superiorly when incorporated in structural systems that are prone to collision and impact events. As also stated, the laminated nature of FRPs and the susceptibility of FRPs to delamination, especially invisible delaminations, render the consideration of impact events critical and necessary in designs that utilize FRPs. Consider a material like aluminum under an impact loading condition. An impact on aluminum will potentially create a large deformation, or in the most critical situation, penetration. In the case of most metallic alloys, such outcomes would potentially be non-detrimental to the overall integrity of the impacted structural components due to the ductility of most metals and the consequential plastic deformation. Most metals also offer a strain-hardening effect, thereby offering an additional degree of sustenance to such events—an attribute not shared with most laminated FRP composites.

In an event where an FRP is subjected to a suddenly applied load, the loading will develop localized interlaminar shear and normal tensile stresses, in turn causing damage to the composite since most FRPs lack any through-thickness reinforcement. These stresses are usually the initial causes of structural failure on the microscopic level. For this reason, design failure strains are usually as low as 0.5% [29,30]. This low design failure strain results in an FRP with significantly underutilized in-plane material strength. Depending on the velocity of the applied load, the loading could be categorized into low-, high-, and hyper-velocity categories. At low velocities, the interaction between the material and the impactor is relatively long enough for the effect of the event to extend beyond the zone of impact. Cantwell and Morton [29] classified LVI as an impact event that takes place below 10 m/s. This value gives a good approximation for most materials, whereas Robinson and Davies [31] outlined a more accurate method by considering the through-thickness stress wave and its effects, concluding that the stress-wave dominant effects start taking place between 10 and 20 m/s [13]. A recent study also explored new high-performance impact-resistant FMLs made with the more resilient Elium 188 resin, UHMWPE fibers, and titanium alloy [32]. Like the other impact studies noted above, this study also showed the superior energy absorption capacity of the composite. In addition, they observed the FML dissipated impact energy through several different failure mechanisms such as fiber breakage, resin cracking, metal plastic deformation, and delamination at the metal/resin/fiber interface. However, they observed the severe effect of LVI on the compression-after-impact resistance of the composite.

In general, the failure modes observed as a result of an impact event are categorized into three categories, as follows:

- Delamination mode, which would occur when there existed a strong difference gradient in the bending stiffness between fiber layups [33]. Such delaminations would have an oblong appearance, with the longer axis being parallel to the fiber direction [34]. Dorey [34] stated that the development of delamination would be more likely in composites with a shorter length than longer. This observation, combined with that of Liu [33], is believed to be the most critical combination causing a delamination.
- Matrix cracking mode mainly occurs when the absorbed energy leads to the formation of micro- and macrocracks. Unlike in delamination, matrix cracking occurs across fibers. This effect usually occurs when the impact energy is within a smaller range of values, usually below 5 J. It should be noted that damage to the matrix is usually the first form of damage during an impact event and is usually located in planes parallel to the fiber direction, caused by the existence of a differential gradient between the properties of the fiber and matrix [12]. These cracks are commonly categorized into two categories: (a) bending cracks and (b) shear cracks [35]. Bending cracks are usually formed perpendicular to the fibers and originate between fiber layers at the boundary of the fiber–matrix interface, whereas shear cracks, which are usually oriented at a 45-degree angle to the fibers, are formed as a result of large traverse shear stress often resulting from an impact [35].
- Penetration mode occurs under concentrated high-impact energy levels, resulting in significant damage to both the matrix and the fibers. The significant damage to the matrix forces the fibers to sustain the load. Cantwell and Morton [36] found that the mode of penetration that has the highest energy absorption is the shear-out failure. This failure mode is recognized by a plug of material sheared out of the composite panel, absorbing between 50 and 60% of the impact energy, depending on the thickness of the composite.

As previously noted, most load-bearing FRPs are made of thermoset resin, primarily epoxies, which are generally brittle and can sustain relatively low failure strains. As a result, the full fiber strength is usually underutilized, which affects the feasibility of composites in many applications. It is worth mentioning that Sela and Ishai [37] found that improvement in the fracture toughness of resin can lead to the application of failure strain values 50% higher than those commonly used with epoxies. They also demonstrated that the use of thermoplastic resins like poly-ether-ether-ketone (PEEK) improved the fracture toughness of their composite by an order of magnitude, though this resulted in poor fiber–resin interface bonding.

Similar to an LVI event, HVI plays a critical role in engineering designs involved with high speeds (e.g., aviation, automotive, space, and speed boat industries). In an HVI event, the effect of stress wave dominates the event. The velocity domain at which stress-wave effects become dominant can be established by using a simplified equation like that suggested by Robinson and Davies [31]. Olsson found that during an impact event, there exists a continuum of three wave types [38]. Three-dimensional dilatational waves dominate impact events where the event duration approaches the through-thickness wave propagation period [38,39]. As the time of the impact event increases, the waves transition to flexural waves until a quasi-static state is achieved. Olson also discovered that the quasi-static waves were affected by the size of the target and the boundary conditions, but these parameters did not influence the dilatational, flexural, and shear waves [39]. It should be noted that the failure modes described earlier for LVI events also hold for HVI events. The major difference between the two is in an LVI event: one mode usually dominates the failure mode, whereas in HVI events, a mixture of the modes is generally experienced.

Another parameter that significantly affects the ability of composites to absorb energy during HVI is the angle at which the impactor strikes the target; the larger the angle, the more adverse the outcome would be [40]. Siva Kumar and Balakrishna Bhat [40] concluded that a small increase in the angle of impact had minimal effect on the energy absorbed, but once the angle of incident surpassed 30 degrees, an increase in the absorbed energy would be observed. The modes of penetration have a further delineation depending on the material and its properties. During an impact event, a compression wave is generated. If this wave exceeds the compression strength of the material, radially spaced cracks will occur, which is common in materials where the compression strength is greater than the tensile strength [41,42].

**Figure 1:**
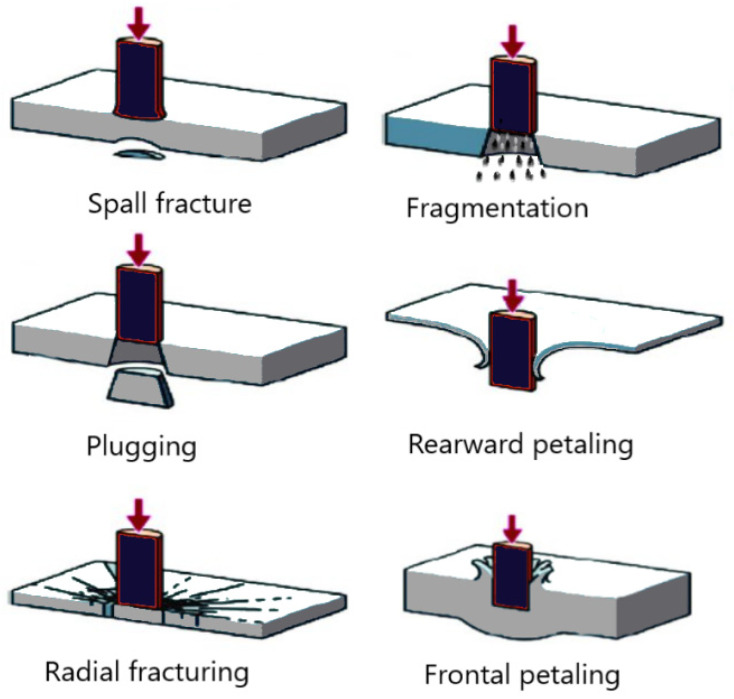
HVI damage modes.

Another well-known failure mode is referred to as “petaling” (see Figure 1), which occurs when a high amount of tensile stress is developed at the back side of an impacted FRP panel during an event and is released once the damage has occurred. Another phenomenon known as “fragmentation”, also shown in Figure 1, is caused by the localized pulverization of the material upon impact and is seen more prominently in brittle materials [41,42]. Finally, “plugging” is where a cylindrical portion of the impacted material is ejected during an impact event, which is caused by a high amount of through-thickness shear stress development around the borders of a blunt projectile [41,42]. This damage mode is illustrated in Figure 1.

From the design perspective, an important metric in HVI is the “ballistic limit”, which represents the velocity at which a material is penetrated. This value varies for each material and configuration. Extensive work has been conducted in developing predictive models for both composites and other common engineering materials [43,44]. These models have been shown to produce somewhat reasonable approximations; however, the accuracy is not adequate for engineering applications [43] Ferriter et al. [43] investigated the effectiveness of the following models for establishing the ballistic limit: bisection; the Jonas-Lamber method; the “Source velocity versus Received velocity method” or “Vs vs. Vr”; the Golden Ratio; the Residual Energy vs. the angle of the projectile relationship; and the residual energy vs. receiving velocity, Vr, relationship. They concluded that the bisection method produced the lowest error margin (i.e., 1 m/s) in a test using a sample size of five specimens.

The projectile geometry has a significant influence on the modes of failure in HVI [44]. Mines et al. [45] found that the impactor with a hemispherical geometry had the highest target energy absorption for stitched fabric, while the flat impactor had the highest energy absorption for the woven fabric. They concluded that flat impactors would cause material failure mainly due to punching shear, while the round and conical impactors would cause a mixed failure mode involving tensile, shear, and bending stresses. Conical impactors would also generate a mixed failure mode of tensile and shear failure modes.

## 3. Materials and Methods

### 3.1. Reinforcing Fibers

As stated earlier, four different fibers are used in this study to fabricate the FRPs examined in this investigation, namely E-glass, aramid (specifically, Kevlar 29), and basalt fibers.

The Kevlar fabric used in this research was a fabric mat with unidirectional fiber orientation and an areal density of 320 g per square meter, produced by the Dupont™ company (acquired from Albarrie Canada, Barrie, ON, Canada). Kevlar is an organic fiber classified within the family of aromatic polyamides [46]. Since its discovery, Kevlar has been incorporated into several engineering applications in many industries, ranging from aviation to defense. Due to its molecular orientation, this fiber provides good resistance against wear and impact loading.

The E-glass fiber fabric used in this research was a biaxial 0/90 stitch mat fabric with an areal density of 427 g per square meter, supplied locally by Burnside Fiberglass (Dartmouth, NS, Canada).

The basalt-reinforcing fabric used in this research was also a biaxial (0/90) fabric with an areal density of 516 g per square meter. The fabric was supplied by GBF Basalt Fiber Co., Ltd. (Dongyang, Zhejiang, China). The mechanical properties of the above fibers, obtained from the vendors, are reported in Table 1.

### 3.2. Resins

#### 3.2.1. Elium^©^ 150

The Elium 150 resin system used in this research was supplied by Arkema Inc., (King of Prussia, PA, USA). It came in two parts: part A, the Elium resin, and part B, the curative agent (Arkema’s Luperox organic peroxide), which are mixed at a recommended ratio of hardener to resin by weight of 1.5–3% and have a maximum pot life of 30 min [48]. In this research, the ratio of 2% was used as the ratio yielded the best performance through trial and error.

The resin is fully cured at room temperature in 24 h; this period can be reduced to as low as 3 h by increasing the temperature during curing. The resin will begin to turn a pink hue once the curing process has begun and then will transition to a transparent resin upon full curing. The mechanical properties of the resin, provided by the producer, are listed in Table 2.

#### 3.2.2. West Systems Epoxy

The two-part room-cured epoxy system is produced by West Systems (Bay City, MI, USA). The system has very low chemical volatility. The mix ratio varies based on the hardener used (fast, slow and extra slow); in our case, a slow hardener was used with a resin-hardener ratio of 5:1 [49]. When implementing this resin, the processes that can be utilized are not limited to vacuum-assisted processes as the crosslinking process will take place in an open environment.

## 4. Fabrication of the Composites

Vacuum-assisted resin infusion molding (VARTM) was used to produce the FRP panels, which, compared to vacuum-bagging and hand-layup methods, provides more consistent part quality. Moreover, it facilitates the low-oxygen environment Elium requires, with a more controllable fiber volume ratio. The FRP panels were fabricated using a resin-to-fiber weight ratio of 1:1. The vacuum-bag assemblies were left to cure under vacuum for 24 h. The readers are referred to [50] for a detailed description of fabrication details of the composite with Elium resin.

It should be noted that after considering our hands-on experience with Elium resin, we found that achieving a sound composite requires a higher level of expertise compared to using epoxy. Even determining the appropriate percentage of the hardener (Luperox organic peroxide), which the resin manufacturer recommends is within a range of 1.5% to 3%, required extensive testing to ensure a workable fabrication period for the panels. Fabrication with Elium necessitates an oxygen-minimized environment, demanding specific manufacturing conditions and considerable skill. Given our laboratory’s somewhat limited facilities and our familiarity with Elium, we opted for the 1:1 ratio, somewhat an arbitrary ratio, which through trial and error provided a reasonable timeframe for panel fabrication. Consequently, we maintained this ratio across all composites to ensure consistency. All panels had a [0/90]_2,s_ layup.

## 5. Experimental Investigations

### 5.1. Evaluation of the Basic Mechanical Properties

The basic mechanical properties of the composites were evaluated using the standard test methods as per ASTM. Tensile, compressive, and shear tests were conducted according to ASTM D3039, D3410, and D3518; the tensile and compressive tests were conducted on specimens with a [0/90]_2,s_ layup; and the shear tests were performed on specimens with a [+45/−45]_2,s_ layup. The tensile test cross-ply layup was used based on the fact that the LVI and HVI tests were conducted on cross-ply FRPs, which are commonly used in practical applications; therefore, it was concluded that the basic properties should also be evaluated on the same layup. Moreover, when a biaxial FRP with a relatively high fiber volume is subjected to tensile or compressive loading, a great percentage of load (>95%) of the load is sustained by the longitudinally oriented fibers. Finally, the compressive strength of a biaxial FRP can be calculated with great accuracy using the “back-out” factor, as documented in MIL-HDBK-17-F [51,52].

The appropriate size specimens were extracted from the glass and basalt panels using a water-cooled diamond-coated rotary saw, whereas the Kevlar composite specimens were extracted using a water-jet cutting machine since the aramid composite could not be cut by the rotary saw. The dimensions of the tensile and shear specimens were 250 (L) × 25 (W) × 2.5 (t) mm following the standards [53,54].

To establish the specimen length (or, specifically, the gauge length) used in compression testing, Equation (1), provided in ASTM 3410 [55], was used. The use of the equation ensures that the specimens would not undergo premature buckling.

(1) $$h\geq \frac{l_{g}}{0.9069\sqrt{\left(1-\frac{1.2F^{cu}}{G_{xz}})(\frac{E^{c}}{F^{cu}}\right)}}$$

where $l_{g}$ is the ga1uge length, $F^{cu}$ is the ultimate compressive stress, $G_{xz}$ is the through-thickness modulus, and $E^{c}$ is the longitudinal modulus of elasticity. As can be seen, the use of the equation requires the selection of some mechanical properties that have to be guessed—or better, can be estimated based on the rule of mixture. The calculated approximate thickness of the composites was between 3.5 and 4 mm, yielding compression specimen dimensions of 145 (L) × 25 (W) × 4 (t) mm.

**Figure 2:**
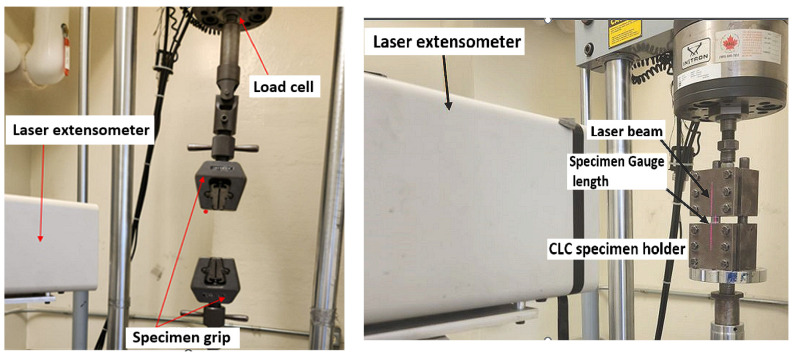
The test setups for (**left**) tensile test and (**right**) compression test.

The specimens were tested using a digitally controlled MTS servo-hydraulic universal test system. The strain in the gauge length was measured using a laser extensometer (Model LE-05, by Electronic Instrument Research, Irwin, PA, USA), shown in Figure 2. This extensometer utilizes two retro-reflective pieces of tape to reference the distance during a testing event. The setup for a typical compression test is shown in Figure 2. The compression specimen is restrained by the combined loading compression fixture (CLC) fixture as per ASTM D3410 [55].

Once the test data was processed, the mechanical properties were evaluated based on the basic mechanics of materials equations noted in each ASTM standard.

### 5.2. Impact Tests

#### 5.2.1. Low-Velocity Impact Test

The experimental setup for the LVI test implements a modified Charpy impactor. The impactor arm is manually raised to the desired angle, which is pre-calibrated to the required energy level. Once the arm is at the desired angle, the brake lever at the pivot is engaged, holding the arm in place. Once the brake is released, the arm swings forward, impacting the impactor contact point on the linear impactor propulsion guide. The linear impactor propulsion guide is a jig consisting of a series of roller bearings that restrict the impactor travel in all degrees of freedom except for translation in the direction of impact. It is positioned so that the linear direction extending from the arc of the impactor at the point of contact forms a tangent line.

**Figure 3:**
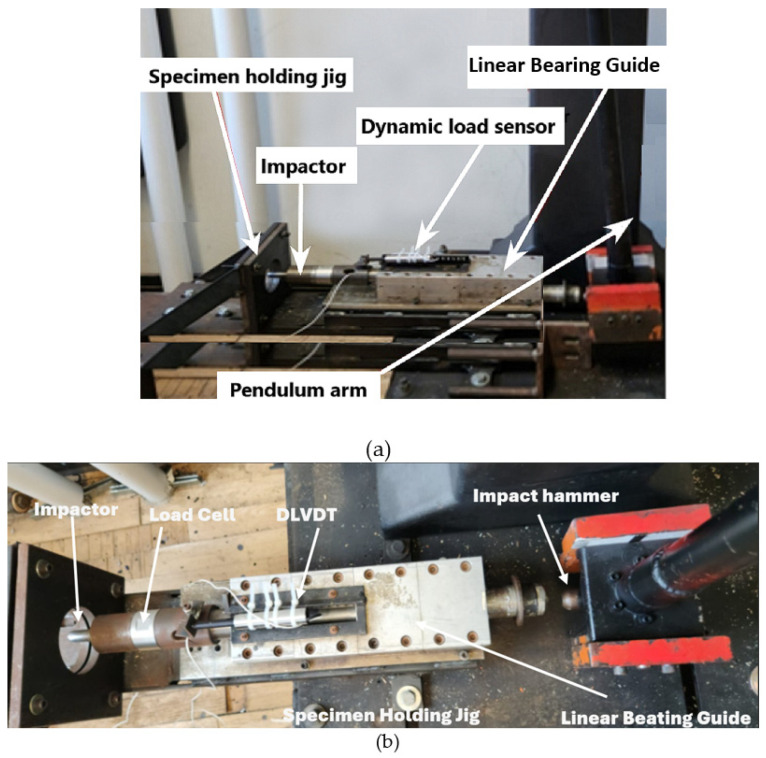
Low-velocity impact test setup (**a**) front view; (**b**) top view.

The impactor tip used in this experiment is a hemispherical impactor, with the whole impactor assembly having a mass of 5.822 kg. The deformation-measuring device implemented for the low-velocity setup is a dynamic linear variable differential transformer (DLVDT). The obtained values of deformation or displacement versus load are collected and analyzed, showing the indentation depth in the specimen. The force response of the impact event is measured using a Dytran 1060 dynamic load cell. This load cell is placed before the impactor so that during an event, the compression force of the impact is measured. The signals from the DLVDT and the load cell are processed using LabVIEW, and the final data are saved into a text file. The specimen holder is constructed of two steel plates that clamp the specimen, with a circular opening to restrain the specimen during the impact, which is bolted rigidly to the main rigid platform. The setup details can be seen in Figure 3.

To ensure that each specimen is restrained in a uniform axisymmetric fashion, the specimen-holding jig was made to have a circular opening with a diameter of 80 mm instead of a square opening. A circular opening facilitates even restraint, thus ensuring even stress distribution at the boundaries by the holder. The setup was precisely calibrated. Details of the calibration procedure for the LVI testing equipment can be found in our earlier study [56].

#### 5.2.2. High-Velocity Impact Test

**Figure 4:**
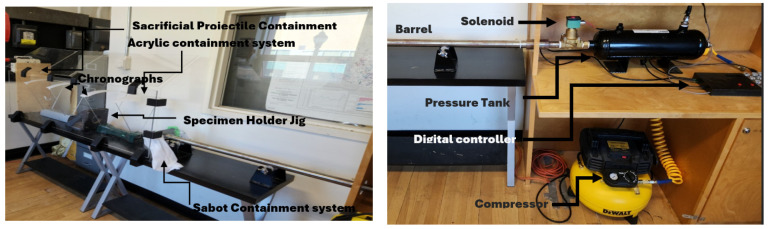
High-velocity test setup.

The experimental setup for the HVI test utilizes a compressed-air gas gun, designed and fabricated in-house. It consists of a pressure gas chamber (which holds air or helium if a higher speed is desired), a barrel, an electro-mechanical solenoid valve, and a digital microelectronic system controlled by Arduino code and an air compressor, as shown in Figure 4. A breach is located at the rear end of the 25 mm diameter barrel, which accommodates the loading of the projectile, held in a specially designed sabot. The microcontroller controls the operation of the propelling mechanism. The projectiles are propelled using an electro-mechanical solenoid valve that is closed when uncharged but opens once charged. Compressed air was used as the propellant gas as it could accommodate the maximum values of energy required; however, a helium cylinder is also available if higher energies are required.

**Figure 5:**
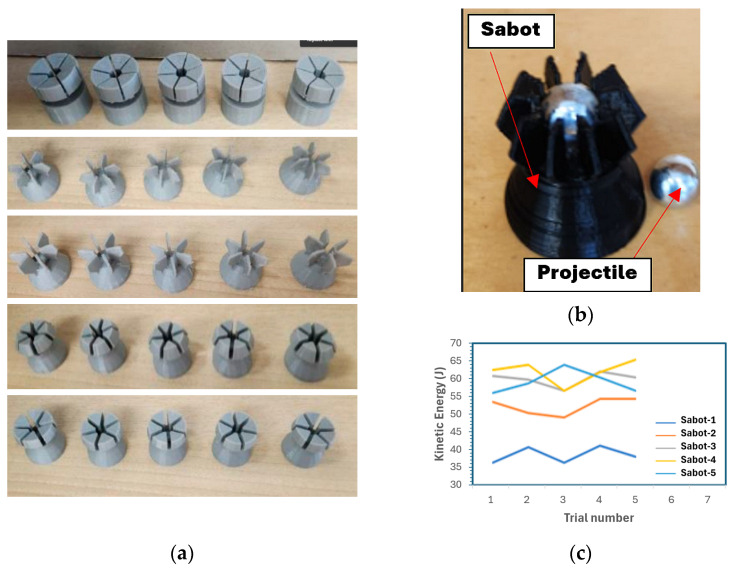
(**a**) Designed sabots; (**b**) performance of the five designed sabots; (**c**) final sabot hosting a projectile.

A 9.53 mm diameter solid stainless-steel ball was used as the projectile based on the rationale discussed in [44]. As stated, the propulsion of the ball was facilitated by an in-house designed sabot. Five different designs were developed, 3D-printed, and tested. The five designs are illustrated in Figure 5a. Although some may look quite alike, the minute differences in dimension and appearance resulted in significantly different performances, as shown in Figure 5b. The sabot facilitates the efficient use of the propellant by maintaining a proper seal with the barrel’s internal wall. The sabot is then ejected from the projectile through the use of a sabot arresting system. The shattered sabot arresting system consisted of a muzzle deflector that would engage the projectile release mechanism designed into the sabot, allowing the projectile to continue forward while the sabot redirected into a catching system. The pressure gas tank has a manufacturer-rated maximum pressure of 1.38 MPa (200 psi). Given the high velocities the system is capable of, the testing area is encapsulated within a protective shielding system. This system consists of 12 mm thick plexiglass side walls with 5 mm thick plexiglass angled top covers. The sacrificial backing panel is composed of plywood panels with a total thickness of 60 mm, as this material provides good stopping capabilities while reducing the chance of a ricochet.

The velocity systems used in this experimental setup are two ballistic chronographs and a digital and analog pressure gauge. The two chronographs are located before and after the test specimen. This placement allows for the measurement of the velocities and energies of the projectile before and after an impact event. The precision ballistic chronographs are manufactured by Caldwell and Competition Electronics. They are capable of measuring velocities between 1 and 3000 m/s with a manufacturer-rated accuracy of ±0.25% and ±0.5%, respectively [57,58]. This level of accuracy is achieved through the implementation of 48 MHz processors. The specimen dimensions used in the experimental setup are 100 mm × 150 mm, as recommended by ASTM D7136 [59]. The specimen-holding device consists of two plates that sandwich the test specimen. The holding jig is then bolted rigidly to the testing platform.

The HVI tests were performed following the US Army Research Laboratory’s penetration [43]. This method is called the v50 method and is based on the principle that there exists a function that describes the behavior of the projectile after impact and penetration [43]. First, a range velocity is selected which should include the limit velocity. For this experiment, given that the range of the gas gun propellant speed is somewhat limited, the entire velocity range of the gas gun was selected. The specimen is placed and centered into the holding jig. Next, the chronograph, control computer, and air pump are turned on and the projectile is loaded into the breach. Then, the required pressure for the desired velocity is established in the main air tank and the projectile is propelled. The velocity of the projectile is picked up by the chronographs and recorded. If penetration is achieved, then the range is halved, and the process is repeated until the ballistic limit is reached. According to the document, this method can accurately obtain the ballistic limit with a tolerance of 1 m/s with an average of five impact events [43].

## 6. Results and Discussion

### 6.1. Basic Material and Mechanical Properties

#### 6.1.1. Density and Void Content Results

The average fiber contents were evaluated using the burn-off test, and the void content of the tested composites was also established following ASTM D2734-16 [60]. The results are reported in Table 3.

All void contents fall below 8% of the total volume of the composites, with the E-glass–Elium having the highest void content and the Kevlar-29–epoxy having the lowest. The epoxy composite specimens (except for basalt–epoxy) have a lower void content than their Elium counterparts, with the basalt–epoxy being comparable to basalt–Elium. This is partially due to the increased outgassing caused by the rapid increase in temperature caused by the cross-linking of Elium, leading the resin to approach its boiling point. The fiber contents are also similar in value, with Kevlar-29 Elium having the highest fiber volume. The resin occupies most of the volume of a composite because it has the lower density of the two, and the composite is manufactured with an approximately 1:1 resin-to-fiber weight ratio. This ratio is observed with all specimens except with the Kevlar-29–Elium, where the fiber volume is larger than the resin. The Kevlar-29 fibers are closer in density to the resin; therefore, this increase in fiber volume with a decrease in fiber density is expected.

#### 6.1.2. Tensile Test Results

**Figure 6:**
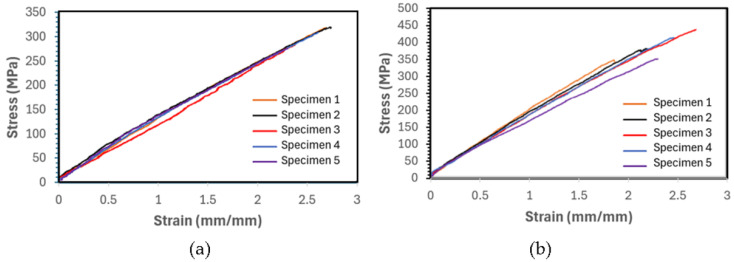
Typical stress–strain curves of (**a**) basalt–epoxy and (**b**) basalt–Elium.

As mentioned earlier, the tensile tests were performed following ASTM D3039 [53] on all composite specimens, with five specimens tested for each composite. The measurements of width, length, and thickness were taken at three different points each, and the averages were recorded. Typical stress–strain curves are shown in Figure 6, and the average results for all composites are summarized in Table 4.

As seen, the ultimate tensile strength of each Elium composite has a higher value than its epoxy-based counterpart, with Kevlar-29–Elium having the highest value of 641.6 MPa. The increase in strength can be explained by looking at the fundamentals of composite mechanics. In composites, the matrix facilitates load transfer among the fibers. In unidirectional fiber-reinforced composites, the fibers primarily take a great majority of the load, even if their volume content is low. However, any deviation in fiber angle with respect to the applied load reduces the load bearing of the fibers, at which state the resin contribution comes into action, with the contribution becoming significantly increased as the fiber angle increases. In the case of our specimens, there are also fibers laid in the transverse directions. With consideration of this phenomenon, one should also consider the ductile nature of Elium compared to the rigid and brittle nature of epoxy resins. Any malalignment during the assembly process, which is a function of the stochasticity of the system, and any void content will have graver consequences in a brittle matrix than a ductile one. This effect can also be observed in the materials recorded modulus of elasticity.

#### 6.1.3. Compression Test Results

**Figure 7:**
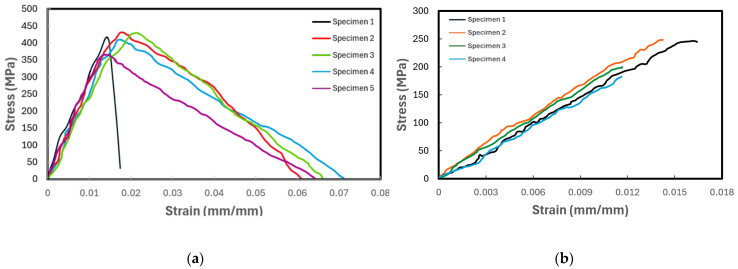
Plot of compressive stress vs. strain of (**a**) E-glass–Elium and (**b**) E-glass–epoxy.

**Figure 8:**
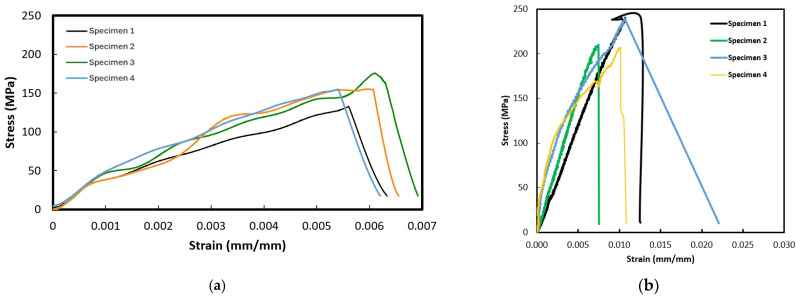
Plot of compressive stress vs. strain of (**a**) basalt–Elium and (**b**) basalt–epoxy.

In this test, a CLC fixture described in detail in ASTM D3410 [55] was used to secure the specimen, with five specimens tested for each composite. The test specimens were prepared with fiber orientations of [0/90]_2,s_. The average specimen thickness for each group of specimens was calculated based on Equation (1) for a set gauge length. It should be noted that since the compressive strength of Kevlar composites is known to be significantly lower than their tensile strength, the testing of Kevlar–epoxy under compression loading was not carried out. The test results in the format of average strength and elastic modulus for the tested composites are tabulated in Table 5, and the stress–strain curves of all tested specimens are illustrated in Figure 7 and Figure 8 for E-glass composites and basalt composites, respectively.

As seen, the differences in the compressive stress–strain curves for each composite are relatively larger compared to those obtained through the tensile tests. This is usually the case in the world of FRPs since any voids of defects would affect the performance of FRPs more significantly than when FRPs are loaded in tension. In a tensile loading, the load is primarily carried out by the fibers, and the presence of void or defects in the resin would have a very minor effect on the performance of the FRP. However, the fiber volume, voids, and defects would have a more significant effect due to the fact that fibers are under tensile stress in the orthogonal to loading direction and through-the-thickness.

Additionally, in the graphs presented in Figure 7 and Figure 8, for the E-glass and basalt composites, respectively, the epoxy-based FRPs exhibit a lower ultimate compressive strength than the Elium-based FRPs. This response is consistent with the results of the tensile tests. The reason for this could be attributed to the ductility of Elium compared to epoxy, in turn mitigating the initiation of microcracks and their propagation and ultimately to shearing and delamination.

#### 6.1.4. Shear Test Results

**Figure 9:**
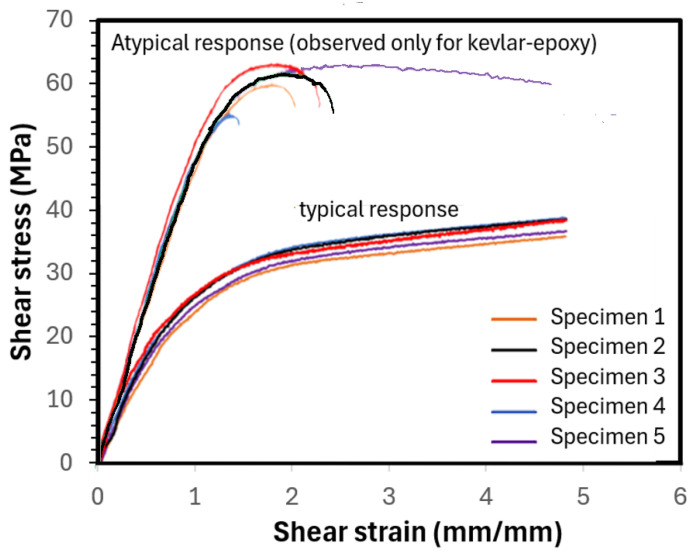
Typical and atypical graphs of shear stress vs. shear strain graphs of the composites.

As stated earlier, the shear tests were performed as per ASTM D 3518 [54], which is quite similar to the tensile test, except in this test, the specimen fiber orientation is +45° and −45°. The fiber orientation is symmetric from the mid-plane. Each test was performed with a sample size of five specimens per test. The results of the test are reported in Figure 9. As seen, the figure shows two sets of shear-stress versus shear-strain behaviors, with both sets following the typical expected nonlinear response. The lower set of curves illustrates essentially typical responses, as observed for all composites except for the Kevlar–epoxy specimens. The atypical response—that is, the limited plastic-like response that most Kevlar–epoxy specimens exhibited—is also shown in the same figure.

As seen from the average results reported in Table 6, the composites with basalt and E-glass fibers and the Elium matrix exhibited a relatively higher shear strength. The higher ultimate shear strength is due in part to the effective transfer of load among the fibers accommodated by the matrix. Elium, as mentioned previously, has a more ductile nature and, thus, can deform more than epoxies before reaching failure. Also, the ductile nature reduces the effect of stress concentration, which otherwise would result in a graver consequence in a brittle matrix. However, the Kevlar-29–Elium did not follow the trend. The reason for this is believed to be due to the comparatively much smaller-diameter (finer) fibers of the fabric compared to E-glass and basalt fabrics. This smaller fiber diameter combined with the low viscosity of the Elium resin at its liquid state is believed to facilitate more resin being absorbed by (within) the fibers. Given that the in-plane shear strength is a strong function of the matrix mechanical properties, more resin within the fibers and less between the bundles would lead to an increase in the effective shear strength. This effect was not observed with the basalt and E-glass composites as the fiber diameters are significantly larger than the Kevlar-29 fibers and are comparatively inherently less absorbent. Moreover, the space between fiber bundles for both the E-glass and basalt is significantly larger than the Kevlar-29. Therefore, the smaller space facilitates the resin placement in between the fiber bundles, leading to a reversed trend, as seen. A similar issue is believed to have caused the less-ductile responses shown by the Kevlar-29–epoxy specimens.

### 6.2. Low-Velocity Impact Test Results

As stated earlier, the LVI test is performed following ASTM D7139 [59] with a few modifications to the setup as also explained in the previous chapter. Briefly, this test was performed using a modified Charpy impacting device. This device is the only modification to the testing setup outlined in ASTM D7139 since the method employed in the standard is a drop-weight testing setup. The use of the modified method eliminates the bouncing back of the falling weight in the drop weight method, thereby eliminating the multi-impacting associated with the falling weight method.

Three levels of impact energy (i.e., 25, 40, and 55 Joules) were used, and three specimens per energy level were tested. The average maximum sustained force per impact energy for all composites is reported in Table 7.

**Figure 10:**
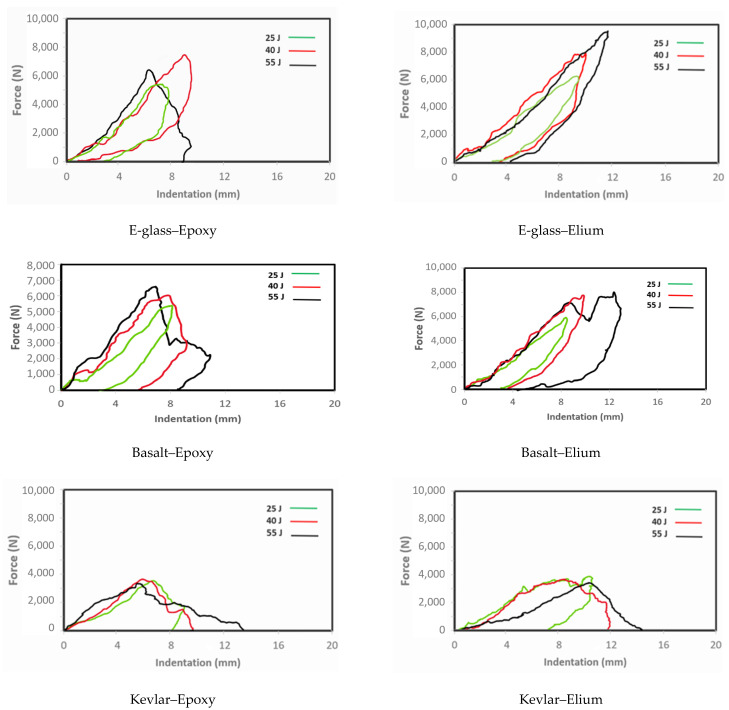
Plot of impact load versus indentation depth of the composites subjected to the three impact energies.

The LVI response of E-glass–epoxy specimens subjected to 55 Joules, as seen in Figure 10, shows a spike in the force during the impact, which is relatively short-lasting (approximately, 0.004 s) due to the penetration experienced by the material. This failure is evident in the indentation depth trace as the indentation increases without reverberation. Additionally, the sharpness of the curves is reduced as the impact energy is decreased to 40 Joules and then to 25 Joules. The indentation depth for both the 40-Joule and 25-Joule responses shows that reverberation has taken place, signifying that a penetration event has not occurred—rather, the material has progressively damaged locally, albeit insignificantly.

The LVI responses of E-glass–Elium specimens subjected to 55-Joule responses, seen in the same figure, show a similar response to the epoxy composites but with a more gradual decrease in force after reaching the peak force. One of the three specimens experienced full penetration, while the other two experienced partial penetration. The failure modes in both epoxy- and Elium-based glass composites were primarily dominated by delamination and fiber tear-out. The results of these impact events, tabulated in Table 7, indicate that Elium-based composites maintained a higher impact force before any deformation occurred compared with the epoxy composites. This trend was observed for all energy levels.

**Figure 11:**
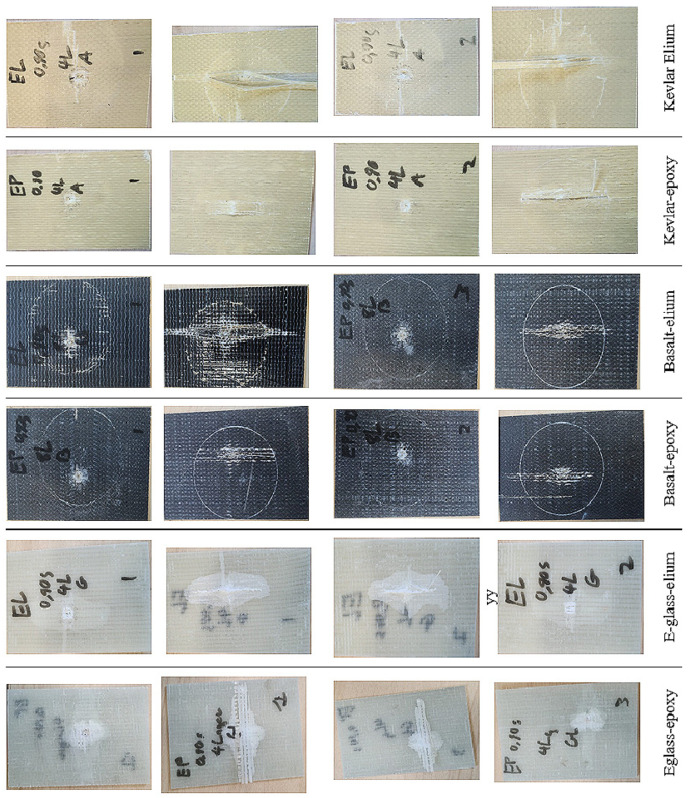
Typical post-low-velocity impact images of the composites (impacted surface on top, reverse non-impacted surface, below).

The basalt–epoxy specimens exhibited a similar response to the E-glass–epoxy. The sudden and abrupt nature of the impact event can be seen in the figure, signified by the abrupt increase and decrease in force as the event progresses. The curves indicate partial and full penetration accompanied by mild reverberation. As can also be seen, the sharpness of the curves is reduced at lower impact energy. The post-impact visuals shown in Figure 11’s images provide evidence that the major failure modes observed are similar to the E-glass, with delamination and fiber tear-out being the most prominent modes. A different response can be observed when comparing the post-impact pictures of the representative specimens of basalt–epoxy and basalt–Elium composites. It can be observed that the characteristic sharp response in the curves is not projected in the observed damage modes. Looking at the photos in Figure 11, one sees no evidence of full penetration, even under 55-Joule impact energy. The observable major failure modes are local deformation and mild delamination. This deformation is characteristic of the plastic deformation of a ductile material.

The Kevlar–epoxy composite’s impact responses shown in Figure 10 illustrate a more gradual response, however, exhibiting significantly lower capacity than both the E-glass and basalt–epoxy composites. The indentation traces provide insight into this observed effect. Every specimen, regardless of the impact energy, experienced penetration, except, surprisingly, for one of the specimens subjected to 55 J energy. The comparison of the load–indentation curves of epoxy- and Elium-based Kevlar specimens in Figure 10 shows that at the higher energy levels (55 and 40 J), the specimens were penetrated without reverberation. However, the pertinent sub-figures shown in Figure 11 show no evidence of any penetration. As seen, the Kevlar–Elium composite specimens underwent significant local, permanent, nonlinear deformation in larger areas compared to all other composites, thus absorbing most of the applied energy without compromising the structural integrity of the fibers within the composite. This local nonlinear deformation explains why the indentation trace occurred the way it did, whereas mild reverberation is observed in the case when the composite was subjected to 25-Joule impact.

### 6.3. High-Velocity Impact Test Results

As stated earlier, the HVI test is performed following the US Army Research Laboratory Bisection Method [43], using a compressed-air gas gun projecting a 9.53 mm diameter steel spherical projectile with a mass of 3.5 g. Six specimens were used per composite material configuration. The velocities before and after were recorded using the two chronographs and are reported.

The results reported in Table 8 reveal that the Elium-based composites generated higher values of the limit velocity compared to the epoxy-based composites in all cases. This higher ballistic limit is again attributed to the more ductile nature of Elium and its higher fracture toughness compared to epoxy. The higher ductility and fracture toughness facilitate the absorption of more energy compared to the brittle counterpart. This increased energy absorption is based on the fact that the area under the stress–strain curve signifies the energy that can be absorbed by a material or the strain energy capacity of the material. A ductile material has a larger area as it has a curve that extends past its linear elastic region (or past its yield strength). The same is not true for brittle materials, as brittle materials tend to have a linear region with minimal or no strain-carrying capacity beyond the yield point.

The results in the table also evidence that the use of Elium combined with Kevlar produced the greatest enhancement in ballistic performance compared to its epoxy-based counterpart. The next-highest enhancement is exhibited by basalt. The results also show that basalt–epoxy outperformed E-glass–epoxy. This higher performance is worth noting since basalt is a naturally occurring substance, and its pairing with a recyclable plastic can see potential applications, potentially replacing E-glass–epoxy composites as a more environmentally friendly alternative.

Furthermore, the normalization of the ballistic limit velocity reported in Table 8 better shows the superiority of the Elium-based composites, outperforming their epoxy counterparts. The normalized results confirm the superiority of basalt amongst the fibers tested. However, while the energy absorption of Elium composites also surpasses their epoxy-based counterpart, the energy absorption of Kevlar–Elium is superior to basalt–Elium.

**Figure 12:**
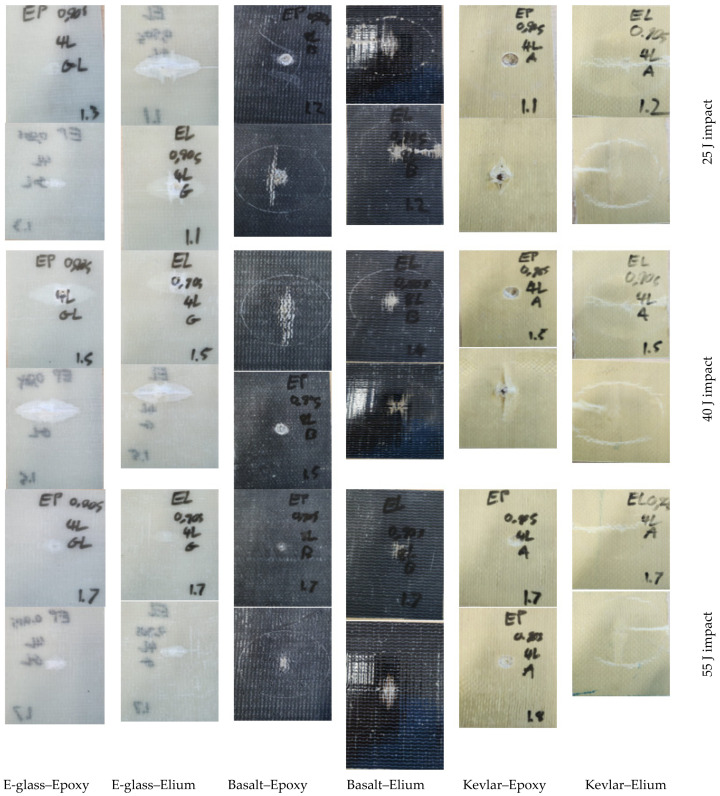
Typical post-impact damage modes of all composite types tested under low-velocity impact. Each row illustrates the front and reverse side of the specimens, sequentially.

The typical representative post-impact failure mode images of all specimen categories are illustrated in Figure 12. In these figures, the impacted surfaces are placed above the reverse (or non-impacted) face of the specimens. As can be seen, the epoxy-based composites’ failure modes are dominated by fiber pull-out, fiber tear-out, and delamination. The most dominant fiber pull-out mode is observed in the cases of both the E-glass and basalt-based epoxy composites, while the fiber tear-out and delamination seem to be less significant contributors. For the Kevlar-29 composites, the most common failure mode is the fiber tear-out, where the tear-out event is primarily localized to the point of penetration. Conversely, the failure modes of Elium-based counterparts are more fiber tear-out, fiber pull-out, delamination, and local deformation. The Kevlar-29 composites have similar failure mode profiles as the basalt composites.

Moreover, the Elium composites show more prevalent local deformation due to Elium’s ductile nature. It is worth noting that while the similarities in failure modes of delamination, fiber pull-out, and fiber tear-out are common in both epoxy-based and Elium-based composites, the characteristics of these modes differ. In other words, the fiber pull-out, fiber tear-out, and delamination seem to have sharper borders, with a more apparent inflexible protrusion for the epoxy-based composites, whereas the Elium composites exhibit a rounded and flexible nature in these modes.

## 7. Summary and Conclusions

The primary objective of this study was to characterize and compare the mechanical performance, including low- and high-velocity impact responses, of six different fiber-reinforced composites. These composites were made using three types of fibers (E-glass, Kevlar, and basalt) and two types of resins (epoxy and Elium). The motivation behind this research was to expand the limited dataset for Elium-based composites, particularly those reinforced with basalt fiber. The ultimate aim was to evaluate the feasibility of basalt–Elium composites, which are mineral-based, fully recyclable, environmentally friendly, and cost-effective for various structural applications.

The following conclusions were drawn from the investigation:

- All composites exhibited an average void content of approximately 7% and similar average fiber weight percentages, except for Kevlar–Elium, which had slightly higher fiber weight content.
- Elium composites demonstrated superior tensile responses compared to epoxy composites.
- Shear property evaluations revealed that both E-glass and basalt–Elium-based composites displayed higher ultimate shear strength and modulus values compared to their epoxy-based counterparts, with E-glass–Elium exhibiting the highest values. However, this trend was not observed in Kevlar composites.
- Compressive property evaluations yielded mixed results: E-glass–Elium outperformed its epoxy counterpart, whereas basalt–epoxy composites outperformed basalt–Elium composites.
- Elium-based composites exhibited significantly better low-velocity impact responses compared to epoxy-based composites. Basalt–Elium demonstrated the highest energy absorption capacity, with epoxy composites displaying more brittle failure modes and higher degrees of penetration during impact events. Kevlar-29–Elium experienced significant nonlinear and irreversible deformation under the highest impact energy, remaining unpenetrated. Some basalt–Elium specimens also remained unpenetrated, with smaller surface indentations compared to Kevlar–Elium.
- High-velocity impact tests yielded results consistent with low-velocity impacts. Elium-based composites outperformed epoxy-based composites in both tests, with basalt–Elium achieving the highest ballistic limit. Similar failure modes were observed between low- and high-velocity tests, with Elium-based specimens exhibiting higher elastic-plastic deformation, while epoxy composites displayed brittle failure modes such as matrix cracking and projectile punch-through.

## Figures and Tables

**Table 1:** Manufacturer-supplied material properties of the various fibers used in this study.

Fiber	Tensile Strength (MPa)	Tensile Modulus (MPa)	Density g/(cm^3^)	Elongation at Break (%)	Reference
Kevlar-29	3600	83,000	1.44	3.60%	[46]
E-glass	3400	72,000	2.54	4.70%	[47]
Basalt	4840	89,000	2.70	3.15%	[3]

**Table 2:** Manufacturer-supplied material properties of a 4 mm unfilled cured Elium 150 resin casting [48] and West Systems epoxy [49].

Property	Elium 150	West Systems Epoxy
Shore D Hardness	85–90	83
Tensile Strength	76 MPa	50 MPa
Tensile Modulus	3300 MPa	3172 MPa
Tensile Deformation	6%	4.5%
Flexural Strength	130 MPa	81 MPa
Flexural Modulus	3250 MPa	3103 MPa
Compression Strength	130 MPa	79 MPa
Cured Specific Gravity	1.19	1.18
Liquid Viscosity	100 mPa·s	725 mPa·s

**Table 3:** Average values of composites density, void content and fiber volume fraction.

Material	Average Density (g/mL)	Void Content %	Average Fiber Volume Fraction	Average Resin Volume Fraction
E-glass–Epoxy	1.49	4.68	0.29	0.66
E-glass–Elium	1.35	7.04	0.29	0.64
Basalt–Epoxy	1.59	6.54	0.32	0.61
Basalt–Elium	1.49	5.51	0.32	0.63
Kevlar–Epoxy	1.24	4.19	0.43	0.53
Kevlar–Elium	1.15	6.42	0.49	0.45

**Table 4:** Average tensile properties of the composites.

Material	E-Glass–Epoxy	E-Glass–Elium	Basalt–Epoxy	Basalt–Elium	Kevlar-29–Epoxy	Kevlar-29–Elium
Tensile Strength (MPa)	301.5 (23.4) *	304.9 (2.33)	298.5 (18.2)	386.8 (27.4)	463.2 (11.4)	641.6 (9.8)
Tensile Modulus (GPa)	11.073 (0.647)	12.424 (0.475)	13.854 (0.725)	16.566 (0.996)	27.828 (0.775)	35.044 (0.894)

* The values in brackets represent the standard deviations.

**Table 5:** Average compressive properties of the composites.

Material	E-Glass–Epoxy	E-Glass–Elium	Basalt–Epoxy	Basalt–Elium
Compressive Strength (MPa)	218.25 (30.4) *	419.96 (15.18)	223.67 (16.07)	146.51 (11.8)
Compressive Modulus (GPa)	16.720 (0.533)	27.232 (2.91)	23.930 (3.515)	27.779 (9.09)

* The values in brackets represent the standard deviations.

**Table 6:** Average shear strength and modulus of test specimens.

Material	E-Glass–Epoxy	E-Glass–Elium	Basalt–Epoxy	Basalt–Elium	Kevlar-29–Epoxy	Kevlar-29–Elium
Shear Strength (MPa)	47.9 (1.78) *	69.8 (1.82)	36.8 (1.16)	39.9 (2.67)	61.3 (2.82)	11.2 (0.66)
Shear Modulus (GPa)	3.145 (0.198)	4.475 (0.273)	2.677 (0.130)	3.365 (0.097)	4.221 (0.197)	1.269 (0.063)

* The values in brackets represent the standard deviations.

**Table 7:** Variation of impact force as a function of impact energy for the composites.

Material	Impact Energy (J)	Average Force (N)	Standard Deviation
E-glass–Epoxy	55	7114.85	527.07
	40	7511.58	44.96
	25	5982.90	735.46
E-glass–Elium	55	8373.15	466.14
	40	7892.95	260.80
	25	6631.65	443.14
Basalt–Epoxy	55	7165.22	617.11
	40	6886.80	244.49
	25	6029.54	88.67
Basalt–Elium	55	8831.26	518.70
	40	7942.06	718.62
	25	6484.40	511.77
Kevlar–Epoxy	55	3051.58	670.51
	40	4115.38	222.93
	25	3648.67	196.26
Kevlar–Elium	55	3497.20	41.13
	40	3885.79	52.61
	25	4634.42	517.83

**Table 8:** Ballistic limit velocity and energy absorption capacity of the composites.

Material	Ballistic Limit Velocity—BLV (m/s)	Comparative * Energy Absorption Capacity (J)	% Increase in * Energy Absorption Capacity	Normalized BLV with Respect to Glass–Epoxy (m/s)
E-glass–Epoxy	128.5	-	-	1
E-glass–Elium	131	1.14	1.95	1.02
Basalt–Epoxy	142.5	-	-	1.11
Basalt–Elium	148	2.80	3.86	1.15
Kevlar–Epoxy	116	-	-	0.90
Kevlar–Elium	122	2.50	5.17	0.95

* The values are calculated with respect to their epoxy-based counterparts.

## Data Availability

The data presented in this study are available on request from the corresponding author and upon the approval of Arkema (for the Elium-related date). The data are not publicly available due to restrictions under a non-disclosure agreement (NDA) with the material provider, which limits the sharing of specific data outside of pre-approved parties.
